# Comparative genomic profiling of glandular bladder tumours

**DOI:** 10.1007/s00428-020-02787-8

**Published:** 2020-03-20

**Authors:** Angela Maurer, Nadina Ortiz-Bruechle, Karolina Guricova, Michael Rose, Ronja Morsch, Stefan Garczyk, Robert Stöhr, Simone Bertz, Reinhard Golz, Henning Reis, Felix Bremmer, Annette Zimpfer, Sabine Siegert, Glen Kristiansen, Kristina Schwamborn, Nikolaus Gassler, Ruth Knuechel, Nadine T. Gaisa, for the German study group of bladder cancer

**Affiliations:** 1 Institute of Pathology, University Hospital RWTH Aachen University, Pauwelsstrasse 30, 52074 Aachen, Germany; 2 Department of Urology, University Hospital RWTH Aachen University, Aachen, Germany; 3 Institute of Pathology, University Hospital Erlangen, Erlangen, Germany; 4 Institute of Pathology, HELIOS Clinic Wuppertal, Wuppertal, Germany; 5 Institute of Pathology, University Hospital Essen, University of Duisburg-Essen, Essen, Germany; 6 Institute of Pathology, University Medical Center, University of Göttingen, Göttingen, Germany; 7 Institute of Pathology, University Medical Center Rostock, Rostock, Germany; 8 Institute of Pathology Munich-North, Munich, Germany; 9 Institute of Pathology, University Hospital Bonn, Bonn, Germany; 10 Institute of Pathology, Technical University Munich, Munich, Germany; 11 Institute of Legal Medicine, Section Pathology, University Hospital Jena, Jena, Germany

**Keywords:** Bladder adenocarcinoma, Urothelial carcinoma with glandular differentiation, Urachal carcinoma, Urothelial carcinoma, Molecular genetics

## Abstract

Primary glandular bladder tumours (bladder adenocarcinoma [BAC], urachal adenocarcinoma [UAC], urothelial carcinoma with glandular differentiation [UCg]) are rare malignancies with histological resemblance to colorectal adenocarcinoma (CORAD) in the majority of this subgroup. Definite case numbers are very low, molecular data are limited and the pathogenesis remains poorly understood. Therefore, this study was designed to complement current knowledge by in depth analysis of BAC (*n* = 12), UAC (*n* = 13), UCg (*n* = 11) and non-invasive glandular lesions (*n* = 19). In BAC, in addition to known alterations in TP53, Wnt, MAP kinase and MTOR pathway, mutations in *SMAD4*, *ARID1A* and *BRAF* were identified. Compared to published data on muscle invasive bladder cancer (BLCA) and CORAD, UCg exhibited frequent “urothelial” like alterations while BAC and UAC were characterised by a more “colorectal” like mutational pattern. Immunohistochemically, there was no evidence of DNA mismatch repair deficiency or PD-L1 tumour cell positivity in any sample. Depending on the used antibody 0–45% of BAC, 0–30% of UCg and 0% UAC cases exhibited PD-L1 expressing tumour associated immune cells. A single BAC (9%, 1/11) showed evidence of ARID1A protein loss, and two cases of UCg (20%, 2/10) showed loss of SMARCA1 and PBRM1, respectively. Taken together, our data suggest at least in part involvement of similar pathways driving tumourigenesis of adenocarcinomas like BAC, UAC and CORAD independent of their tissue origin. Alterations of *TERT* and *FBXW7* in single cases of intestinal metaplasia further point towards a possible precancerous character in line with previous reports.

**Electronic supplementary material:**

The online version of this article (10.1007/s00428-020-02787-8) contains supplementary material, which is available to authorized users.

## Introduction

Primary adenocarcinoma of the bladder is a rare malignancy accounting for < 2% of all bladder cancers [1]. Thus, in Germany, less than 200 cases of adenocarcinoma are expected each year (about 16.400 new bladder cancer cases in 2016) [2]. Besides pure primary bladder adenocarcinoma (BAC), glandular and mucinous differentiation of bladder tumours (1.6% or 0.8% of invasive high-grade tumours) can be found as a sign of de-differentiation in high-grade urothelial tumours (UCg; urothelial carcinoma with glandular differentiation) [3, 4]. Additionally, urachal adenocarcinomas (UAC; tumours arising from embryonic urachal remnants) also present as glandular or mucinous adenocarcinomas. Due to their special origin and different treatment strategies, they are usually considered separately. BAC can exhibit various phenotypes: enteric/colonic, mucinous/colloid, signet-ring cell, clear cell, hepatoid, mixed and adenocarcinoma not otherwise specified (NOS; if without a specific glandular growth pattern) [5]. This phenotypical diversity turns them into diagnostically challenging tumours, since—first of all—metastatic carcinomas must be excluded [6].

So far, the pathogenesis of BAC remains poorly understood, and morphological resemblance to colorectal adenocarcinomas (CORAD) suggests potential analogies. Next-generation sequencing (NGS) data have improved our knowledge of genetic driver alterations in urothelial carcinomas [7] and CORAD [8], and first NGS data are now available for rare BAC [9, 10] and UAC [11–16]. Additionally, a few single gene sequencing reports for BAC and UAC (e.g. *BRAF*, *EGFR*, *KRAS*, *NRAS*, *PIK3CA*, *TERT*) have been published [17–21]. Due to these studies alterations in “urothelial” (e.g. *RB1*) as well as “colorectal” (e.g. *APC*, *KRAS*), associated genes have been identified for BAC and UAC. An involvement of MAP kinase, MTOR, Wnt and TP53 pathway in BAC [9] and UAC [13] has been described.

However, these previous studies did not comparatively analyse BAC, UCg, UAC and possible precancerous glandular lesions (cystitis glandularis [CG] and intestinal metaplasia [IM]) in parallel to reveal specific tumourigenic events and pathways for each entity.

Therefore, the aim of our study was to decipher genomic similarities and differences in glandular bladder tumours (BAC, UAC and UCg) in comparison to publicly available data on muscle invasive urothelial cancers (BLCA) and CORAD using a custom NGS panel covering all exons of 20 urothelial and colorectal driver genes in order to understand tumour biology and reveal suitable (targeted) therapeutic concepts. Additionally, tumours were screened for *TERT* promoter mutations and analysed immunohistochemically for DNA mismatch repair (MMR) deficiency, loss of SWI/SNF complex expression and PD-L1 expression.

## Materials and methods

### Patient samples and tissue microarray construction

Formalin-fixed, paraffin-embedded (FFPE) archival bladder cancer specimens from ten different Institutes of Pathology in Germany were collected. Each case was carefully checked within the pathology archives/data bases and by cross-check with the referring urologists in a three-step process to verify correct classification and exclude metastatic tumours (see Supplementary Methods 1 for further information). Tumour classification was performed according to the 2017 International Union Against Cancer [22] and the 2016 World Health Organization classification of bladder tumours [5]. In total, *n* = 12 BAC (*n* = 9 enteric, *n* = 2 mucinous and *n* = 1 mixed morphology); *n* = 13 UAC (*n* = 10 mucinous and *n* = 3 enteric); and *n* = 11 UCg, *n* = 3 CG and *n* = 1 IM were available for analysis with confirming clinical data, sufficient material for sequencing and appropriate sequencing data for successful single nucleotide variants (SNV) and copy number alteration (CNA) analysis. In addition, *n* = 8 CG and *n* = 7 IM samples with low material were analysed only with *SNapShot*® for *TERT* promoter mutations. Tissue microarrays were constructed as previously described [6]. Clinico-pathological data of the patient cohort are shown in Table 1 and of each patient individually in Supplementary Table 1. The retrospective, anonymous study was approved by the local Ethics Committee (EK 286/11).

**Table 1:** Clinico-pathological data of patient cohort

	Bladder adenocarcinoma (*n* = 12)	Urachal adenocarcinoma (*n* = 13)	Urothelial carcinoma with glandular differentiation (*n* = 11)
Patient age (years)			
30–49	1	7	2
50–69	4	5	3
70–89	7	1	6
Gender			
Female	3	6	3
Male	9	7	8
Tumour stage			
pT1	6	–	4
pT2	2	–	3
pT3	2	–	4
Tx	2	2	0
TIIIA	–	6	–
TIIIB	–	4	–
TIIIC	–	1	–
Tumour grade			
G1	0	1	0
G2	10	10	2
G3	2	2	9
Nodal status			
N0	1	8	1
N1	0	0	2
Nx	11	5	8
Subtype			
Enteric	9	3	–
Mucinous	2	10	–
Mixed	1	0	–

### Microdissection and DNA isolation

For microdissection, five to 15 freshly cut serial FFPE sections (4 μm) were deparaffinised and stained with 0.1% methylene blue. Using a stereo microscope, areas with tumour cells were collected manually with sterile needles. DNA isolation was performed by using QIAamp™ DNA Mini Kits (Qiagen, Hilden, Germany) according to the manufacturer’s instructions.

### Targeted next-generation sequencing

For NGS, a self-designed amplicon panel (TruSeq Custom Amplicon v1.5, Illumina, San Diego, CA, USA) was used covering all coding exons of 20 genes known to be frequently mutated in either BLCA or CORAD (*APC*, *ARID1A*, *BRAF*, *CDKN1A*, *CDKN2A*, *CTNNB1*, *FBXW7*, *FGFR3*, *HRAS*, *KDM6A*, *KRAS*, *MSH6*, *NRAS*, *PIK3CA*, *PTEN*, *RB1*, *SMAD4*, *STAG2*, *TP53*, *TSC1*). Library preparation was performed according to the manufacturer’s protocols, and sequencing was conducted on a MiSeq® benchtop sequencer (Illumina). Raw data were processed directly on the MiSeq (MiSeq Control Software, v2.6, Real-Time Analysis software, v1.18.54). For alignment and variant calling, the SeqNext Module of the Sequence Pilot software (version 4.4.0, JSI medical systems GmbH, Ettenheim, Germany) was utilized. All non-synonymous variants with a frequency of 10% and a coverage of at least 200× were considered for further analysis. To exclude potential germline variants, variants with an allele frequency > 1% in public population databases (gnomAD, [23]) were removed prior to manual review of the remaining variants. Additionally, all oncogene hotspots (RAS: Codon 12, 13, 59, 61, 117, 146; *CTNNB1*: Codon 33-45, *BRAF*: Codon 600, *PIK3CA*: Codon 545, 1047, *FGFR3*: 11 activating mutations) were examined for sufficient coverage, and hotspot variants with a frequency of > 5% were added to the variant list.

High-level CNAs were identified from amplicon coverage data with a recently developed algorithm, based on the efficiency of PCR exponential growth of single amplicons in all measured samples (ACopy, [24]). For visualisation of variants, oncoprints were created with OncoPrinter on http://cbioportal.org [25, 26].

### SNaPshot® analysis for TERT and FGFR3 mutations

*SNapShot*® Multiplex System assay (Applied Biosystems, Foster City, USA) was used to simultaneously screen for 11 known activating *FGFR3* point mutations (R248C, S249C, G372C, S373C, Y375C, G382R, A393E, K652E, K652M, K652Q and K652T, [27]) and for *TERT* promoter mutations at positions -124 (C228T) and -146 (C250T) [28, 29].

### Immunohistochemical analysis of DNA mismatch repair proteins, SWI/SNF complex and PD-L1

TMAs were stained for DNA mismatch repair proteins (MLH1, MSH2, MSH6, PMS2), programmed death-ligand 1 (PD-L1) and SWI/SNF complex components (SMARCB1, SMARCA2, SMARCA4, PBRM1, ARID1A) to assess protein expression. A detailed description of the utilized staining methods, antibodies and scoring systems are found in Supplementary Methods 2–4.

## Results

### Genomic alterations in glandular bladder tumours

**Fig. 1:**
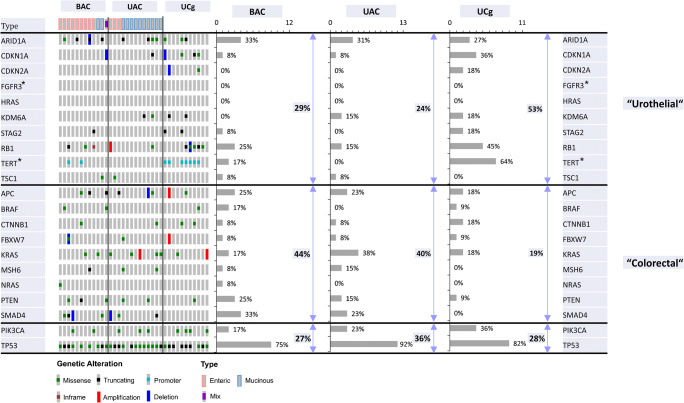
Genomic alterations in glandular bladder tumours. Non-synonymous variants (missense, truncating, inframe and promoter mutations) and CNA (amplifications and deletions) of 21 genes with mutation frequencies for each gene in each subgroup are shown (*only hotspots analysed with *SNaPshot*®). Overall, 36 glandular bladder tumours were analysed (*n* = 12 BAC, *n* = 13 UAC and *n* = 11 UCg). Additionally, cumulative frequencies for alterations of “urothelial” or “colorectal” associated genes are depicted for each subgroup

DNA of 36 glandular bladder tumours (12 BAC, 13 UAC and 11 UCg) was successfully sequenced and analysed for SNVs and CNAs. BAC mainly exhibited an enteric type (9/12) while most UAC showed a mucinous histology (10/13). Clinico-pathological data of the patient cohort are listed in Table 1 (for more detailed data see Supplementary Table 1). Since all oncogenic hotspots except for *FGFR3* were sufficiently covered, 11 activating *FGFR3* mutations were additionally sequenced with *SNaPshot*® analysis. All detected presumably somatic alterations for BAC, UAC and UCg are summarised in Fig. 1. Only one of the analysed samples (UAC, mucinous type) showed no alteration. All other samples harboured between 1 and 9 different changes (all identified SNVs and CNAs are listed in Supplementary Tables 2 and 3). Most frequent alterations in all three subgroups were SNV and CNA of *TP53*, *ARID1A*, *RB1*, *KRAS* and *PIK3CA* (Fig. 1). Additionally, *SMAD4* was altered in BAC (33%, 4/12) and UAC (23%, 3/13), but not in any of the UCg samples. On the other hand, *TERT* promoter mutations were present in 64% (7/11) of UCg cases but only in two (17%, 2/12) BAC cases (both enteric type) and no UAC sample (0/13). All detected *TERT* mutations were located at position -124 (C228T). Due to the low number of analysed cases, a correlation of identified alterations with either mucinous or enteric morphology was not feasible. We detected two mutations of *CTNNB1* (1/12 BAC; 1/13 UAC) and six mutations of *APC* (3/12 BAC; 2/13 UAC; 1/11 UCg); however, *CTNNB1* mutations were not common activation hotspot mutations and no nuclear β-catenin staining was detected in subsequent immunohistochemistry (see Table 2).

**Table 2:** ß-Catenin protein expression and *APC* and *CTNNB1* mutations

Sample	ß-Catenin staining (nucleus)	*APC* mutations	*CTNNB1* mutations
AE-1	na		
AE-2	Negative		
AE-3	Negative		
AE-4	Negative		
AE-5	Negative		
AE-6	Negative	22% G502E	24% E53K
AE-7	Negative		
AE-8	Negative	86% E1573*	
AE-9	Negative		
AM-1	Negative		
AM-2	Negative		
AEM-1	Negative	38% S1465fs	
UM-1	Negative		
UM-2	Negative		
UM-3	Positive		
UM-4	Negative		
UM-5	Negative		
UM-6	Negative		
UM-7	Negative		
UM-8	Negative	28% M485I	
UM-9	Negative		28% W383G
UM-10	Negative		
UE-1	Negative		
UE-2	Negative		
UE-3	Negative	86% K1199*	
UCg-1	na		
UCg-2	Negative		
UCg-3	Negative		
UCg-4	Negative		
UCg-5	Negative		
UCg-6	Negative	66% V2630I	
UCg-7	Negative		
UCg-8	Negative		
UCg-9	Negative		
UCg-10	Negative		
UCg-11	Negative		

*na* not available

**Fig. 2:**
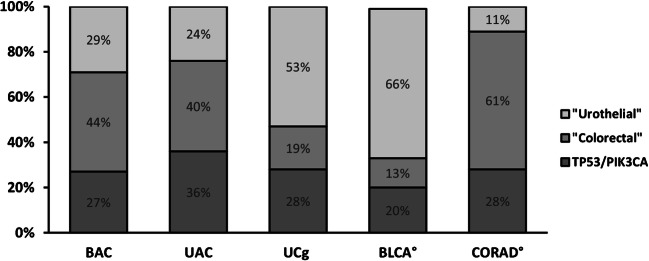
Comparison of cumulative frequencies of alterations in “urothelial” or “colorectal” genes between glandular bladder tumours, BLCA and CORAD. Calculated cumulative alteration frequencies for BAC, UAC, UCg, BLCA and CORAD for ten “urothelial” (*ARID1A*, *CKN1A*, *CDKN2A*, *FGFR3*, *HRAS*, *KDM6A*, *STAG2*, *RB1*, *TERT*, *TSC1*), nine “colorectal” (*APC*, *BRAF*, *CTNNB1*, *FBXW7*, *KRAS*, *MSH6*, *NRAS*, *PTEN*, *SMAD4*) and two additional genes commonly altered in both (*TP53*, *PIK3CA*). °Data for BLCA and CORAD alterations for the 21 genes was obtained from The Cancer Genome Atlas Research Network (TCGA) pan-cancer analysis project (accessed through http://cbioportal.org, [30])

Next, analysed genes were categorised in three subgroups, i.e. an” urothelial” group with ten genes frequently altered in BLCA, a colorectal group with nine genes known to be affected in CORAD and a third group with only two genes (*TP53* and *PIK3CA*) which are both commonly mutated in both tumour entities. UCg showed higher frequencies for alterations in BLCA associated urothelial genes (e.g. *TERT*, *RB1*, *STAG2*, *KDM6A*, *CDKN1A*, *CDKN2A*, *ARID1A*) while BAC and UAC exhibited genomic alterations in colorectal genes (e.g. *KRAS*, *SMAD4*, *PTEN*, *APC)* as well as in urothelial genes (e.g. *ARID1A*, *RB1*). These results can be quantified through calculation of cumulative frequencies for alterations of each of the three groups for BAC, UAC and UCg (Fig. 1) confirming a high participation of urothelial genes in UCg genesis (53%) and an involvement of both urothelial and colorectal genes in BAC (29% vs. 44%) and UAC (24% vs. 40%) development. Additionally, we determined such frequencies for BLCA and CORAD utilising publicly available SNV and CNA data from The Cancer Genome Atlas Research Network (TCGA) (*n* = 406 BLCA, *n* = 526 CORAD, accessed through http://cbioportal.org, [30]). The individual alteration frequencies for BLCA and CORAD for all 21 genes are shown in Supplementary Figure 1. Comparison of these cumulative frequencies for glandular bladder tumours with BLCA and CORAD (Fig. 2) visualises the similarities between UCg and BLCA confirming the above identified urothelial mutational pattern of UCg while proposing a distinct genetic subgroup for BAC and UAC involving urothelial and colorectal aspects.

### DNA mismatch repair enzyme expression and immunohistochemical evaluation of the SNF/SWF complex activity in glandular bladder tumours

Microsatellite instability indicated by DNA mismatch repair enzyme deficiency is well known in CORAD and less frequent in BLCA. Neither one of the analysed BAC (0/12), UAC (0/11) nor UCg (0/9) cases showed a loss of MLH1/PMS2 or MSH2/MSH6 expression (Supplementary Table 3).

**Fig. 3:**
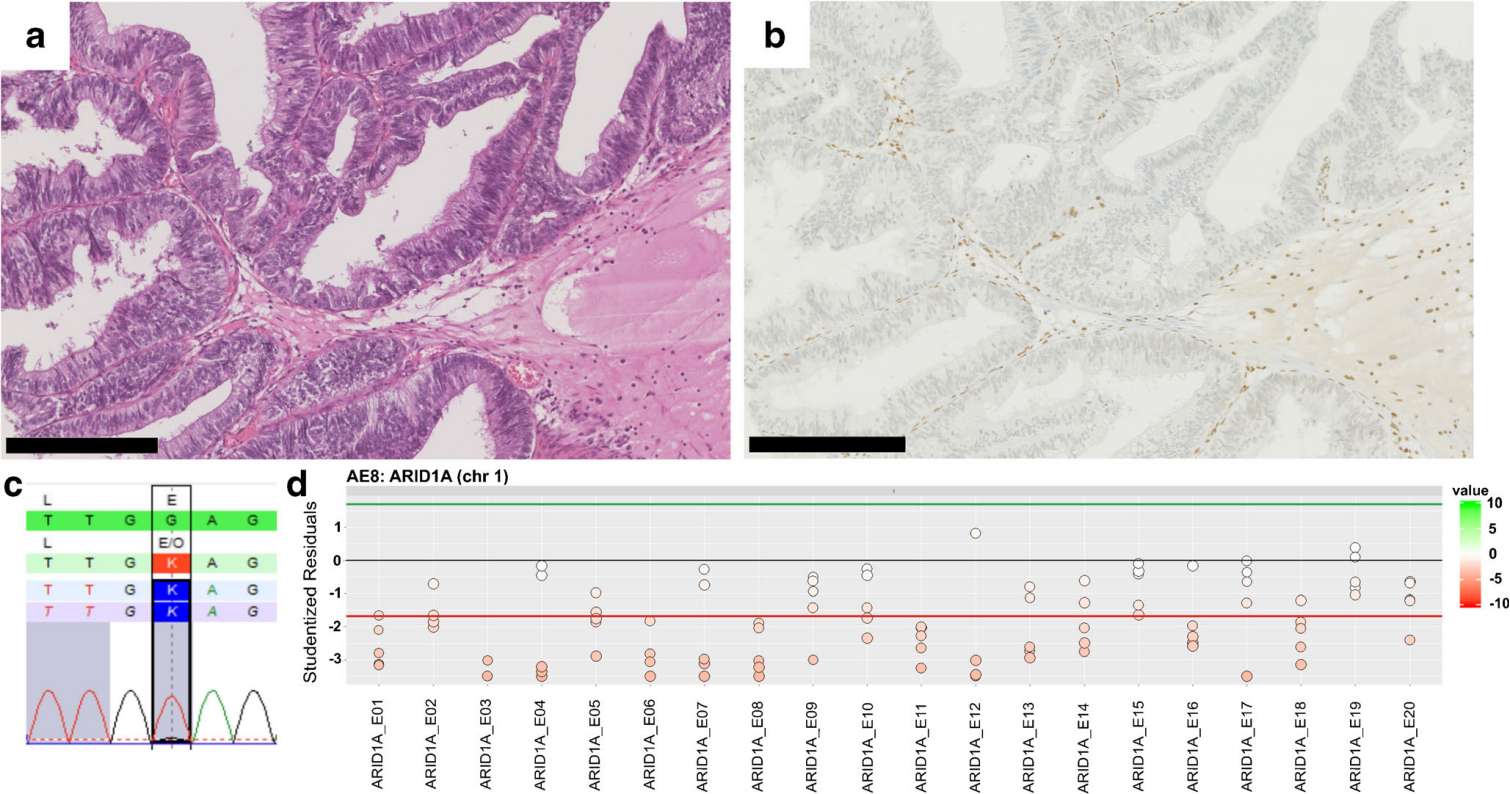
Enteric BAC with loss of ARID1A. HE (**a**) and anti-ARID1A (**b**) staining of a case of BAC (enteric type) with loss of ARID1A expression in tumour tissue (black scale bar equals 250 μm). **c** Truncating *ARID1A* mutation with an allele frequency of 88% (c.6160G>T, p.Glu2054*, estimated tumour content 80%). **d** Relative coverage for all exons of *ARID1A* showing a deletion for sample AE-8. These results were derived through calculation of the relative coverage deviation of each amplicon from the coverage of five correlated amplicons of the same sample. In a normal diploid state with two copies, no deviation in coverage would be detected (= 0)

By analysing the expression of five subunits of the SWI/SNF complex (INI1/SMARCB1, SMARCA2, SMARCA4, ARID1A, PBRM1), we further explored the relevance of alterations in chromatin remodelling in glandular differentiated tumours. One BAC sample (9%, 1/11) exhibited loss of ARID1A expression (Fig. 3b) associated with a truncating *ARID1A* mutation and additional loss of the non-mutated allele in the tumour tissue (Fig. 3c and d). Two UCg samples (20%, 2/10) showed loss of SMARCA1 and PBRM1 respectively, while for two evaluable UAC, no evidence of expression loss of any of the tested markers was detected (Supplementary Table 3).

### PD-L1 expression in glandular bladder tumours

Since immune checkpoint inhibitors (ICI) have been recently approved for treatment of advanced bladder cancers with the necessity of PD-L1 “positivity” in a first-line setting, analysis of PD-L1 expression in glandular bladder cancer might reveal a treatment option for these rare subtypes. Due to the known heterogeneous performance of currently available anti-PD-L1 antibodies [31], all available samples (12 BAC, 3 UAC and 10 UCg) were stained with four different anti-PD-L1 antibody clones (28-8, SP142, SP263, 22C3). Overall, no tumour cell staining (defined as TPS ≥ 1) was observed and none of the three tested UAC showed an immune cell (IC) staining. Depending on the used antibody in 0–45% BAC and 0–30% UCg cases, PD-L1-expressing immune cells were detected (BAC: 3/12 [28-8], 0/12 [SP142], 5/11 [SP263], 3/10 [22C3], UCg: 2/10 [28-8], 0/10 [SP142], 3/10 [SP263], 1/9 [22C3]) with up to three BAC (25%) and three UCg (30%) cases exhibiting an IC-Score above the current threshold for 1st-line atezolizumab therapy in metastatic bladder cancer (IC-Score ≥ 2; Supplementary Figure 2 c and d). Additionally, with obtained CPS (combined positivity score), none of the BAC cases was eligible for 1st-line pembrolizumab therapy while two UCg cases (20%) with an CPS ≥ 10 could be considered (Supplementary Figure 2 a and b). A detailed list of all PD-L1 results (TPS, IC-Score and CPS) for all tested samples and antibodies can be found in Supplementary Table 3.

### Genomic alterations in glandular precancerous lesions

To gain further insights into the development of BAC, we sequenced potential precancerous glandular bladder lesions. Three cases with CG and only one sample with IM were suitable for SNV and CNA analysis with NGS, while the residual cases (*n* = 15) were only sufficient for TERT-*SNapShot*® analysis (clinico-pathological data Supplementary Table 1). Interestingly, in one IM sample, a *TERT* promoter mutation was detectable at position -124 (C228T). All other IM and CG samples displayed *TERT* wildtype in *SNapShot*® analysis. The three sequenced CG cases showed neither oncogenic SNV nor CNA in any of the 20 genes, but in the IM sample, a *FBXW7* alteration (R505G) predicting loss of function was identified (Supplementary Tables 2 and 3).

## Discussion

In this study, we investigated a cohort of glandular bladder–related cancers and non-invasive glandular lesions of the bladder (CG and IM) for genetic profiles. Overall, we assessed the total coding sequence of 20 genes by NGS and additional hotspots of *FGFR3* and *TERT* by *SNapShot*® analysis, in order to compare these profiles with publicly available datasets of BLCA and CORAD. The main questions we wanted to address are the following: (i) are our findings consistent with existing limited data on BAC, UAC and UCg? (ii) are they molecularly related to BLCA or CORAD? (iii) are there molecular events defining preinvasive glandular precancerous lesions? and (iv) are there any distinct therapeutic options for these tumour entities that might improve the current rather organ confined therapeutic regimes?

For rare BAC, currently, only one genomic profiling study (15 samples/51 genes) has been published identifying genomic alterations in genes of MAP kinase, MTOR, Wnt and TP53 pathways [9]. Another study on adenocarcinoma was recently presented but has not yet been published (14 BAC and 10 UAC/275 genes) [10]. Roy et al. described *APC* and *CTNNB1* mutations and nuclear ß-catenin expression (alterations of Wnt signaling) to be involved in BAC development [9]. In line with this study, we detected similar genomic alteration frequencies for *APC* and *CTNNB1*, but we could not show immunohistochemical nuclear ß-catenin translocation, and thus activation of the canonical Wnt pathway cannot be confirmed. We also revealed variants in the Wnt pathway-regulating gene *SMAD4*, which have not been described to be altered in BAC so far. SMAD4 is a tumour suppressor, and transcription factor of the TGF-ß pathway and loss of function alterations have been shown to cause, for instance, impaired response to chemotherapy in colorectal cancer [32]. Downregulation of SMAD4 expression has been identified in pancancer transcriptome analysis to be characteristic for adenocarcinoma independent of origin [33]. However, functional SMAD4 inactivation alone is not sufficient for tumour initiation, but it is thought to promote tumour progression in conjunction with additional alterations, e.g. activating *KRAS* (pancreatic duct adenocarcinoma) or inactivating *APC* alterations (colorectal cancer) [34]. *TP53* was the most frequently altered gene in the study of Roy et al. and ours, and alterations of *FBXW7* (no hotspot variants but cases with loss of function through either mutation or deletion detected) were similar [9]. Previous single gene analyses already identified *KRAS* mutations in BAC [17], which were also present in our cohort (*n* = 2). Roy et al. reported *PIK3CA* mutations as a potential druggable target in BAC [9], which we also confirmed in two samples. Furthermore, we identified two cases with *BRAF* mutations of which one (sample AEM-1) exhibited a hotspot V600E variant. To our knowledge, *BRAF* has not been reported to be altered in BAC before but could represent an important drug target [35]. A key observation of Roy et al. was the absence of any SNV or CNA in BAC with mucinous histology [9]; however, both analysed mucinous BAC cases in our cohort exhibited several mutations in *KRAS*, *TP53* and *ARID1A*, *SMAD4*, *TP53*, respectively. This discrepancy might be due to the low number of analysed cases in both studies (3/15 and 2/12 exhibited mucinous morphology). *TERT* promoter mutations were rarely detected in our study (2/12) in accordance with previously published data (4/14, 0/10 and 2/15 respectively). However, our samples were enteric BAC, whereas Cowan et al. detected *TERT* promoter mutations only in non-enteric BAC and Roy et al. in enteric and non-enteric (single-cell) BAC [9, 18], which are no longer considered to be BAC according to the current WHO classification [36]. In their overview of glandular bladder tumours, Taylor et al. hypothesised that BAC with *TERT* mutation might represent an urothelial subgroup [37]. In our cohort, both *TERT* mutated enteric BAC specimens (AE-3 and AE-6) additionally exhibited colorectal characteristics, i.e. alterations in *SMAD4* and *PTEN*. Taken together, the current results for BAC from our study and from Roy et al. present BAC as a distinct entity exhibiting both characteristics of urothelial (e.g. *TERT* mutations, alterations in chromatin remodelling) and colorectal cancer (e.g. alterations in Wnt pathway) [9].

For UAC, several studies have been published which mainly focus on current therapeutic targets [11–16]. Reis et al. identified, for instance, various druggable alterations (e.g. *BRAF* mutations, single cases of *MET*, *ERBB2* and *EGFR* amplification) while exome-wide studies revealed recurrent alterations in TP53, Wnt/TGF-ß and MAP kinase pathways similar to those detected in our study including 13 UAC samples. In the exome study of Lee et al., UAC samples clustered as a distinct group between BLCA and CORAD comparing CNA profiles [11]. Analogously, our results support this notion as UAC exhibit not only urothelial but also frequent colorectal like alterations. This corroborates the hypothesis that BAC and UAC could be genetically specified as a distinct group between BLCA and CORAD with genetic similarities, although both develop from different sites (urothelium versus urachal remnants) with and without exposure to urine. So far, we are not able to answer the question why site different adenocarcinomas (BAC, UAC) seem to be genetically similar and show overlapping mutational patterns with CORAD including *TP53*, *KRAS* and *SMAD4* [8], as their only similarities are the enteric/goblet cell types. Bearing in mind that SMAD4 function is thought to be a characteristic of adenocarcinomas [33], triggering tumour progression in close association with further mutational drivers such as activating *KRAS*, involvement of comparable molecular pathways driving tumourigenesis of adenocarcinomas like BAC, UAC and CORAD could be suggested independently of the tissue origin.

For UCg—to our best knowledge—there is only a single gene analysis while no genomic profiling studies have been published so far, excluding those analysing BLCA with mixed features (squamous, glandular, etc.) [38]. Vail et al. identified 72% (21/29) of UCg as *TERT* mutated, comparable to our study (64%; 7/11) [39]. Although showing a slightly higher mutational rate in some “colorectal-like” genes (*KRAS*, *APC*, *CTNNB1*) than BLCA, UCg mainly harboured frequent alterations in distinct urothelial-like genes (e.g. *TERT*, *RB1*, *CDKN1A*, *ARID1A* and *KDM6A*) as well as *TP53* and *PIK3CA*. A particularly conspicuous aspect is the high level of *TERT* mutations in UCg which differ from UAC or BAC with only low numbers of TERT mutated cases [40].

We furthermore identified a *TERT* promoter mutation and a missense *FBXW7* variant in one of the tested glandular preinvasive lesions (IM sample). The detected *FBXW7* R505G mutation is located in the WD repeat domain at a recurrently altered hotspot (R505) with R505L and R505C associated with a loss of function through disruption of substrate binding [41, 42]. The tumour suppressor FBXW7 binds to proto-oncogenes mediating degradation, while dysregulation leads to chromosomal instability and tumourigenesis due to accumulation of oncoproteins [43]. In line with previous analysis of *TERT* promoter mutations in glandular bladder tumours including 25 benign glandular lesions of the bladder (with 5 CG samples amongst Brunn nests, cystitis cystica and nephrogenic adenoma), we did not detect any *TERT* variants in the tested CG samples [39]. While a few previous studies also detected neoplastic changes in IM (e.g. telomere shortening and chromosomal abnormalities) suggesting IM to be a precursor of adenocarcinoma, accumulating studies showed coexistence of IM and CG with bladder cancer as well as in benign bladder specimens and no correlation between occurrence of IM and risk for progression to tumour [44–46]. Thus, the debate is still ongoing and further molecular analysis with larger sample numbers and clinical follow-up are needed in order to prove or disprove the precancerous nature of these lesions.

Finally, we assessed our cohort for current predictive immunohistochemical marker expression, i.e. DNA mismatch repair, SWI/SNF complexes and PD-L1. We found no deficiency in DNA mismatch repair enzymes in UCg, BAC and UAC in concordance with the described low frequency of DNA mismatch repair defects in bladder cancer [47] and recent UAC [12] and BAC data [48]. Single SWI/SNF alterations (ARID1A loss in one BAC sample; no alterations in the two analysed UAC samples; two UCg cases with loss of either SMARCA2 or PBMR1 expression) can be found predominantly in the urothelial glandular tumours, with currently no therapeutic consequences [49]. None of the glandular bladder tumours showed PD-L1 expression in tumour cells, but up to 45% (5/11) of BAC and 30% of UCg cases (3/10) showed PD-L1 expression in immune cells; thus, ICI might be a treatment option for a subset of advanced BAC and UCg.

In conclusion, the identified mutational patterns propose not only some molecular similarities but also differences between BAC, UAC and to a certain extent also CORAD, whereas UCg follow a urothelial (BLCA) tumourigenesis. We are aware of the limited sample numbers of these rare tumours in our study; thus, the tumours should be further investigated in larger multi-institutional cohorts especially considering future therapeutic approaches. Additionally, ICI seems to be a reasonable treatment option for a subgroup of BAC and UCg, but less indicated in UAC. Moreover, infrequent molecular alterations of *TERT* and *FBXW7* in IM suggest a possible precancerous character in line with previous rare reports.

## Electronic supplementary material

ESM 1 (DOCX 39 kb)

ESM 2 (DOCX 51 kb)

ESM 3 (DOCX 508 kb)
