# Supplementary material for: Comparative genomic profiling of glandular bladder tumours
Source: Virchows Arch. 2020 Mar 20;477(3):445–54. doi: 10.1007/s00428-020-02787-8 (PMC7443184; doi:10.1007/s00428-020-02787-8)
Supplement: Supplementary file 1 — (DOCX 39 kb) [file 428_2020_2787_MOESM1_ESM.docx]

**Online Resource 1 – Virchows Archiv**

**Supplementary Methods 1-3**

**Comparative genomic profiling of glandular bladder tumours**

Angela Maurer^a^, Nadina Ortiz-Bruechle^a^, Karolina Guricova^a^, Michael Rose^a^, Ronja Morsch^a,b^, Stefan Garczyk^a^, Robert Stöhr^c^, Simone Bertz^c^, Reinhard Golz^d^, Henning Reis^e^, Felix Bremmer^f^, Annette Zimpfer^g^, Sabine Siegert^h^, Glen Kristiansen^i^, Kristina Schwamborn^j^, Nikolaus Gassler^k,l^, Ruth Knuechel^a^, Nadine T. Gaisa^a^ for the German study group of bladder cancer

^a^ Institute of Pathology, RWTH Aachen University, Aachen, Germany

^b^ Department of Urology, RWTH Aachen University, Aachen, Germany

^c^ Institute of Pathology, University Hospital Erlangen, Erlangen, Germany

^d^ Institute of Pathology, HELIOS Clinic Wuppertal, Wuppertal, Germany

^e^ Institute of Pathology, University Hospital Essen, University of Duisburg-Essen, Germany

^f^ Institute of Pathology, University Medical Center, University of Göttingen, Göttingen, Germany

^g^ Institute of Pathology, University Medical Center Rostock, Rostock, Germany

^h^ Institute of Pathology Munich-North, Munich, Germany

^i^ Institute of Pathology, University Hospital Bonn, Bonn, Germany

^j^ Institute of Pathology, Technical University Munich, Munich, Germany

^k^ Institute of Pathology, Hospital Braunschweig, Braunschweig, Germany

^l^ Pathology, University Hospital Jena, Jena, Germany

**Corresponding author:**

Nadine T. Gaisa, MD, PhD; ORCID 0000-0002-4762-3964

Institute of Pathology, RWTH Aachen University

Pauwelsstrasse 30

52074 Aachen, Germany

Phone: +49-241-8089288; Fax: +49-241-8082439; email: [ngaisa@ukaachen.de](mailto:ngaisa@ukaachen.de)

**Supplementary Methods 1** – Verification of correct sample classification and additional immunohistochemistry results used for verification

For correct classification of BAC, UAC, UCg, CG and IM a thorough analysis of all available clinical, histological and immunohistochemical data was conducted. We used a three step evaluation scheme: First, histology of all collected patients was re-cut, stained and re-evaluated by an experienced uropathologist (NTG) and subsequent immunohistochemistry was performed and judged as previously published [1]. Second, pathology reports (tumor localization, specimen surface, resection margins, etc.) and databases (previous samples, clinical information, etc.) of all referring Institutes of Pathology were checked in order to exclude secondary involvement/metastasis. Third, referring urologic surgeons were contacted for clinical history, colonoscopy results and CT/MRI images. Only patients successfully passing the three step evaluation process were further analyzed. Samples with insufficient or contradictory data were excluded.

Selected IHC results are summarized in the following table (used antibodies, staining protocols and scoring for these antibodies used in diagnostic routine were previously described by Broede et al [1]):

| **Sample name** | **CK7**  **(>10% positive)** | **ß-Catenin (nucleus)** | **GATA 3**  **(>10% positive)** | **p63**  **(basal staining)** | **CDX2**  **(>10% positive)** |
| --- | --- | --- | --- | --- | --- |
| **AE-1** | na | na | na | na | na |
| **AE-2** | negative | negative | negative | negative | positive (weak) |
| **AE-3** | positive | negative | negative | negative | positive |
| **AE-4** | negative | negative | negative | negative | positive |
| **AE-5** | negative | negative | negative | negative | positive |
| **AE-6** | positive | negative | positive (60%) | negative | na |
| **AE-7** | positive | negative | negative | na | na |
| **AE-8** | negative | negative | negative | negative | positive |
| **AE-9** | negative | negative | negative | negative | negative |
| **AM-1** | positive | negative | negative | negative | positive |
| **AM-2** | positive | negative | negative | negative | na |
| **AEM-1** | negative | negative | negative | negative | positive |
| **UM-1** | positive | negative | negative | negative | positive |
| **UM-2** | positive | negative | negative | na | positive |
| **UM-3** | positive | positive | negative | na | positive |
| **UM-4** | na | negative | negative | na | positive |
| **UM-5** | negative | negative | negative | na | positive |
| **UM-6** | positive | negative | negative | na | positive |
| **UM-7** | positive | negative | negative | na | positive |
| **UM-8** | negative | negative | negative | na | positive |
| **UM-9** | positive | negative | negative | na | positive |
| **UM-10** | positive | negative | negative | negative | positive |
| **UE-1** | positive | negative | negative | negative | positive |
| **UE-2** | negative | negative | negative | negative (< 10% positive) | positive |
| **UE-3** | negative | negative | negative | negative | positive |
| **UCg-1** | na | na | na | na | na |
| **UCg-2** | positive | negative | positive | negative | negative |
| **UCg-3** | positive | negative | negative | negative | negative (< 10%) |
| **UCg-4** | positive | negative | negative | negative | negative |
| **UCg-5** | negative (5%) | negative | positive | positive | positive |
| **UCg-6** | positive | negative | negative | negative | negative |
| **UCg-7** | positive | negative | positive | negative | negative (< 10%) |
| **UCg-8** | positive | negative | positive | negative | weak positive |
| **UCg-9** | positive | negative | positive | negative | negative |
| **UCg-10** | positive | negative | n.a. | positive | negative |
| **UCg-11** | positive | negative | positive | negative | positive |

na=not available

**Supplementary Methods 2 –** Immunohistochemical analysis of DNA mismatch repair proteins

For DNA mismatch repair proteins heat-induced antigen retrieval of 3 µm TMA sections was performed by PT Link (DAKO, Hamburg, Germany) using Low pH buffer (pH 6, DAKO). After 30 min incubation with primary antibodies (anti-MLH1: clone G168-15, dilution 1:10 (3D Biosciences, Houston, TX, USA); anti-MSH2: clone G219-1129, dilution 1:200 (3D Biosciences); anti-MSH6: clone 44, dilution 1:50 (3D Biosciences); anti-PMS2: clone A16-4, dilution 1:100 (3D Biosciences)) captured epitopes were linked to DAKO EnVisionFLEX™ system and visualised with DAKO EnVisionFLEX™ DAB + Chromogen System in an Autostainer plus (DAKO).

**Supplementary Methods 3** – Immunohistochemical analysis of PD-L1

For expression of programmed death-ligand 1 (PD-L1) slides were stained with four different ready to use antibodies either with Low pH buffer (pH 6 (DAKO); clone 28-8 (DAKO) and clone 22C3 (DAKO)) or High pH buffer (pH 9 (DAKO); clone SP142 (Ventana Medical Systems Inc., Tucson, AZ, USA) and clone SP263 (Ventana Medical Systems Inc.)). PD-L1 expression was scored for tumour cells (TPS, tumour cell proportion score / Cologne-Score), immune cells (IC-Score) and combined (CPS, combined positivity score) as follows: TPS/Cologne Score: 0 = 0 < 1%, 1 = 1 - < 5%, 2 = 5 - < 10%, 3 = 10 - < 25%, 4 = 24 - < 50%, 5 = > 50% [2], IC/Immune cell Score: 0 = < 1%, 1 = 1 - < 5%, 2 = 5 - < 10%, 3 = > 10% [3] and CPS (stained immune cells + stained tumour cells / vital tumour cells *100) according to the PD-L1 IHC 22C3 pharmDx interpretation manual for urothelial carcinoma.

**Supplementary Methods 4** – Immunohistochemical analysis of SWI/SNF complex

For SWI/SNF complex immunohistochemical staining was performed using a fully automated “Benchmark XT System” (Ventana Medical Systems Inc.) and anti-SMARCB1 (INI1: clone MRQ-27, dilution 1:50 (Zytomed, Berlin, Germany)), anti-SMARCA2 (polyclonal antibody, dilution 1:100 (Atlas Antibodies AB, Stockholm, Sweden)), anti-SMARCA4 (clone EPNCIR111A, dilution 1:100 (Abcam, Cambridge, UK)), anti-PBRM1 (clone CL0331, dilution 1:30 (Atlas Antibodies AB) and anti-ARID1A (rabbit polyclonal antibody, ab97995, dilution 1:100 (Abcam)) antibodies according to the manufacturer’s instructions. Positivity was assessed semi-quantitatively according to routine diagnostics (staining intensity 0-3).

**References Supplementary Methods 1-4:**

[1] Broede A, Oll M, Maurer A, Siegert S, Stoerkel S, Golz R, Schwamborn K, Veeck J, Knuechel R, Gaisa NT for the German study group of bladder cancers (2016) Differential diagnosis of bladder versus colorectal adenocarcinoma: keratin 7- and GATA3-positivity in nuclear ß-catenin negative glandular tumours define adenocarcinoma of the bladder. J Clin Pathol 69:307-312. https://doi.org/10.1136/jclinpath-2015-203144

[2] Scheel AH, Dietel M, Heukamp LC, Jöhrens K, Kirchner T, Reu S., Rüschoff J, Schildhaus HU, Schirmacher P, Tiemann M, Warth A, Weichert W, Fischer RN, Wolf J, Büttner R (2016) Prädiktive PD-L1-Immunhistochemie beim nichtkleinzelligen Bronchialkarzinom. Aktueller Stand und Erfahrungen der ersten deutschen Harmonisierungsstudie. Pathologe 37(6):557-567. https://doi.org/10.1007/s00292-016-0189-1

[3] Fehrenbacher L, Spira A, Ballinger M, Kowanetz M, Vansteenkiste J, Mazieres J, Park K, Smith D, Artal-Cortes A, Lewanski C, Braiteh F, Waterkamp D, He P, Zou W, Chen DS, Yi J, Sandler A, Rittmeyer A, POPLAR Study Group (2016) Atezolizumab versus docetaxel for patients with previously treated non-small-cell lung cancer (POPLAR): a multicenter, open-label, phase 2 randomised controlled trial. Lancet 387(10030):1837-1846. https://doi.org/10.1016/S0140-6736(16)00587-0
