# Supplementary material for: Comparative genomic profiling of glandular bladder tumours
Source: Virchows Arch. 2020 Mar 20;477(3):445–54. doi: 10.1007/s00428-020-02787-8 (PMC7443184; doi:10.1007/s00428-020-02787-8)
Supplement: Supplementary file 2 — (DOCX 51 kb) [file 428_2020_2787_MOESM2_ESM.docx]

**Online Resource 2 – Virchows Archiv**

**Supplementary Table 1-3**

**Comparative genomic profiling of glandular bladder tumours**

Angela Maurer^a^, Nadina Ortiz-Bruechle^a^, Karolina Guricova^a^, Michael Rose^a^, Ronja Morsch^a,b^, Stefan Garczyk^a^, Robert Stöhr^c^, Simone Bertz^c^, Reinhard Golz^d^, Henning Reis^e^, Felix Bremmer^f^, Annette Zimpfer^g^, Sabine Siegert^h^, Glen Kristiansen^i^, Kristina Schwamborn^j^, Nikolaus Gassler^k,l^, Ruth Knuechel^a^, Nadine T. Gaisa^a^ for the German study group of bladder cancer

^a^ Institute of Pathology, RWTH Aachen University, Aachen, Germany

^b^ Department of Urology, RWTH Aachen University, Aachen, Germany

^c^ Institute of Pathology, University Hospital Erlangen, Erlangen, Germany

^d^ Institute of Pathology, HELIOS Clinic Wuppertal, Wuppertal, Germany

^e^ Institute of Pathology, University Hospital Essen, University of Duisburg-Essen, Germany

^f^ Institute of Pathology, University Medical Center, University of Göttingen, Göttingen, Germany

^g^ Institute of Pathology, University Medical Center Rostock, Rostock, Germany

^h^ Institute of Pathology Munich-North, Munich, Germany

^i^ Institute of Pathology, University Hospital Bonn, Bonn, Germany

^j^ Institute of Pathology, Technical University Munich, Munich, Germany

^k^ Institute of Pathology, Hospital Braunschweig, Braunschweig, Germany

^l^ Pathology, University Hospital Jena, Jena, Germany

**Corresponding author:**

Nadine T. Gaisa, MD, PhD; ORCID 0000-0002-4762-3964

Institute of Pathology, RWTH Aachen University

Pauwelsstrasse 30

52074 Aachen, Germany

Phone: +49-241-8089288; Fax: +49-241-8082439; email: [ngaisa@ukaachen.de](mailto:ngaisa@ukaachen.de)

**Supplementary Table 1** – Detailed clinico-pathological data of patient cohort

| **Sample name** | **Subtype** | **Gender** | **Age (years)** | **Tumour grade (G)** | **Tumour stage**  **(T)/Sheldon** | **Nodal status (N)** |
| --- | --- | --- | --- | --- | --- | --- |
| **AE-1** | Bladder adenocarcinoma (enteric) | M | 53 | 2 | min. 1 | x |
| **AE-2** | Bladder adenocarcinoma (enteric) | M | 70 | 3 | min. 1b | x |
| **AE-3** | Bladder adenocarcinoma (enteric) | F | 79 | 2 | min. 1 | x |
| **AE-4** | Bladder adenocarcinoma (enteric) | M | 67 | 2 | min. 1 | x |
| **AE-5** | Bladder adenocarcinoma (enteric) | M | 59 | 2 | 3a | 0 (0/27) |
| **AE-6** | Bladder adenocarcinoma (enteric) | M | 70 | 2 | min. 1 | x |
| **AE-7** | Bladder adenocarcinoma (enteric) | M | 84 | 3 | min. 2 | x |
| **AE-8** | Bladder adenocarcinoma (enteric) | M | 38 | 2 | x | x |
| **AE-9** | Bladder adenocarcinoma (enteric) | M | 64 | 2 | x | x |
| **AM-1** | Bladder adenocarcinoma (mucinous) | M | 72 | 2 | 3a | x |
| **AM-2** | Bladder adenocarcinoma (mucinous) | F | 74 | 2 | min. 2a | x |
| **AEM-1** | Bladder adenocarcinoma (enteric-mucinous) | F | 88 | 2 | min.1 | x |
| **UE-1** | Urachal adenocarcinoma (enteric) | M | 34 | 3 | IIIA | 0 (0/13) |
| **UE-2** | Urachal adenocarcinoma (enteric) | M | 57 | 2 | IIIA | x |
| **UE-3** | Urachal adenocarcinoma (enteric) | M | 72 | 2 | x | x |
| **UM-1** | Urachal adenocarcinoma (mucinous) | F | 34 | 2 | IIIA | x |
| **UM-2** | Urachal adenocarcinoma (mucinous) | M | 32 | 2 | IIIB | 0 |
| **UM-3** | Urachal adenocarcinoma (mucinous) | M | 49 | 2 | IIIB | 0 |
| **UM-4** | Urachal adenocarcinoma (mucinous) | F | 38 | 1 | IIIA | 0 |
| **UM-5** | Urachal adenocarcinoma (mucinous) | F | 49 | 3 | IIIB | 0 |
| **UM-6** | Urachal adenocarcinoma (mucinous) | M | 46 | 2 | IIIB | 0 |
| **UM-7** | Urachal adenocarcinoma (mucinous) | M | 66 | 2 | IIIC | 0 |
| **UM-8** | Urachal adenocarcinoma (mucinous) | F | 51 | 2 | IIIA | 0 |
| **UM-9** | Urachal adenocarcinoma (mucinous) | F | 56 | 2 | x | x |
| **UM-10** | Urachal adenocarcinoma (mucinous) | F | 63 | 2 | IIIA | x |
| **UCg-1** | Urothelial carcinoma with glandular differentiation | M | 63 | 3 | 1b | x |
| **UCg-2** | Urothelial carcinoma with glandular differentiation | F | 74 | 3 | 2, CIS | x |
| **UCg-3** | Urothelial carcinoma with glandular differentiation | F | 74 | 2 | min. 1 | x |
| **UCg-4** | Urothelial carcinoma with glandular differentiation | M | 40 | 2 | 3b | 1 (1/27) |
| **UCg-5** | Urothelial carcinoma with glandular differentiation | M | 71 | 3 | 1, CIS | x |
| **UCg-6** | Urothelial carcinoma with glandular differentiation | M | 77 | 3 | min. 2a | x |
| **UCg-7** | Urothelial carcinoma with glandular differentiation | M | 84 | 3 | 2a | x |
| **UCg-8** | Urothelial carcinoma with glandular differentiation | M | 53 | 3 | 3a | 1 |
| **UCg-9** | Urothelial carcinoma with glandular differentiation | M | 77 | 3 | 3 | x |
| **UCg-10** | Urothelial carcinoma with glandular differentiation | F | 39 | 3 | 3a | 0 (0/24) |
| **UCg-11** | Urothelial carcinoma with glandular differentiation | M | 58 | 3 | min. 1 (Bladder exstrophy) | x |
| **CG-1** | Cystitis cystica | M | 51 | - | - | - |
| **CG-2** | Cystitis cystica | M | 41 | - | - | - |
| **CG-3** | Cystitis cystica | M | 33 | - | - | - |
| **CG-4** | Cystitis cystica | M | 28 | - | - | - |
| **CG-5** | Cystitis cystica | M | 41 | - | - | - |
| **CG-6** | Cystitis cystica | M | 58 | - | - | - |
| **CG-7** | Cystitis cystica | F | 56 | - | - | - |
| **CG-8** | Cystitis cystica | M | 81 | - | - | - |
| **CG-9** | Cystitis cystica | M | 54 | - | - | - |
| **CG-10** | Cystitis cystica | M | 41 | - | - | - |
| **CG-11** | Cystitis cystica | M | 45 | - | - | - |
| **IM-1** | Intestinal metaplasia | F | 61 | - | - | - |
| **IM-2** | Intestinal metaplasia | F | 64 | - | - | - |
| **IM-3** | Intestinal metaplasia | M | 28 | - | - | - |
| **IM-4** | Intestinal metaplasia | M | 41 | - | - | - |
| **IM-5** | Intestinal metaplasia | M | 54 | - | - | - |
| **IM-6** | Intestinal metaplasia | M | 41 | - | - | - |
| **IM-7** | Intestinal metaplasia | M | 45 | - | - | - |
| **IM-8** | Intestinal metaplasia | M | 33 | - | - | - |

**Supplementary Table 2** – Detected presumably somatic variants and potential copy number alterations (CNA)

| **Sample name** | **Gene** | **Variant** | **AF** | **CNA** |
| --- | --- | --- | --- | --- |
| **AE-1** | NRAS (NM_002524)  TP53 (NM_00546) | c.181C>A, (p.Gln61Lys)  c.844C>T, (p.Arg282Trp) | 61%  86% | none |
| **AE-2** | ARID1A (NM_006015)  BRAF (NM_004333)  SMAD4 (NM_005359)  TP53 (NM_000546) | c.6659A>G, (p.Asn2220Ser)  c.1780G>A, (p.Asp594Asn)  c.1607T>C, p.(Leu536Pro)  c.993+1G>A, p.? | 36%  33%  27%  25% | none |
| **AE-3** | FBXW7 (NM_033632)  FBXW7 (NM_033632)  PTEN (NM_000314)  RB1 (NM_000321)  RB1 (NM_000321)  SMAD4 (NM_005359)  TP53 (NM_000546)  TP53 (NM_000546) | c.419C>T, (p.Thr140Ile)  c.1079G>A, (p.Arg360Lys)  c.1016C>T, (p.Pro339Leu)  c.1237G>T (p.Glu413*)  c.2106+1G>C, p.?  c.1546C>T, (p.Gln516*)  c.488A>G, (p.Tyr163Cys)  c.672+1G>T, p.? | 27%  30%  29%  45%  40%  21%  64%  32% | FBXW7 deletion |
| **AE-4** | PIK3CA (NM_006218)  TP53 (NM_000546) | c.344G>A, (p.Arg115Gln)  c.817C>T, (p.Arg273Cys) | 21%  82% | SMAD4 deletion |
| **AE-5** | ARID1A (NM_006015) | c.6850del, (p.Gln2284Serfs*23) | 23% | none |
| **AE-6** | APC (NM_000038)  CTNNB1 (NM_001904)  PTEN(NM_000314)  TP53 (NM_000546) | c.1505G>A,.(p.Gly502Glu)  c.157G>A, (p.Glu53Lys)  c.686C>A, (p.Ser229*)  c.772G>A, (p.Glu258Lys) | 22%  24%  52%  65% | none |
| **AE-7** | KRAS (NM_033360)  RB1 (NM_000321) | c.35G>T, (p.Gly12Val)  c.55G>A, (p.Glu19Lys) | 6%  10% | none |
| **AE-8** | APC (NM_000038)  ARID1A (NM_006015)  MSH6 (NM_000179)  TP53 (NM_000546) | c.4717G>T, (p.Glu1573*)  c.6160G>T, (p.Glu2054*)  c.2490del, (p.Leu832*)  c.844C>G, (p.Arg282Gly) | 86%  88%  23%  65% | ARID1A deletion |
| **AE-9** | PIK3CA (NM_006218)  RB1 (NM_000321)  STAG2 (NM_001042750) | c.1633G>A, (p.Glu545Lys)  c.1460_1492del, (p.Leu487_Thr497del)  c.363del, (p.Phe121Leufs*24) | 22%  21%  19% | none |
| **AM-1** | KRAS (NM_033360)  TP53 (NM_000546) | c.35G>T, (p.Gly12Val)  c.713G>T, (p.Cys238Phe) | 42%  50% | none |
| **AM-2** | ARID1A (NM_006015)  ARID1A (NM_006015)  SMAD4 (NM_005359)  TP53 (NM_000546)  TSC1 (NM_000368) | c.554del, (p.Gln185Argfs*47)  c.6835C>A, (p.Leu2279Met)  c.1331A>G, (p.His444Arg)  c.823dup, (p.Cys275Leufs*31)  c.3241C>T, (p.Ser1084Leu) | 18%  32%  50%  22%  18% | none |
| **AEM-1** | APC (NM_000038)  BRAF (NM_004333)  PTEN (NM_000314)  TP53 (NM_000546) | c.4393_4394del, (p.Ser1465Trpfs*3)  c.1799T>A, (p.Val600Glu)  c.518G>A, (p.Arg173His)  c.632C>T, (p.Thr211Ile) | 38%  37%  59%  42% | CDKN1A deletion |
| **UE-1** | KRAS (NM_033360)  TP53 (NM_000546) | c.37G>T, (p.Gly13Cys)  c.818G>A, (p.Arg273His) | 25%  62% | RB1 amplification,  SMAD4 deletion |
| **UE-2** | TP53 (NM_000546)  TSC1 (NM_000368) | c.560-2A>T, p.?  c.256C>T, (p.Arg86Cys) | 40%  30% | none |
| **UE-3** | APC (NM_000038)  PIK3CA (NM_006218)  SMAD4 (NM_005359)  TP53 (NM_000546)  TP53 (NM_000546) | c.3595A>T, (p.Lys1199*)  c.3140A>G, (p.His1047Arg)  c.274C>T, (p.His92Tyr)  c.560-1G>A, p.?  c.869G>A, (p.Arg290His) | 86%  42%  28%  92%  96% | none |
| **UM-1** | ARID1A (NM_006015)  FBXW7 (NM_033632)  MSH6 (NM_000179)  PTEN (NM_000314)  TP53 (NM_000546) | c.2530C>T, (p.Gln844*)  c.1222G>A, (p.Ala408Thr)  c.1867C>A, (p.Pro623Thr)  c.1026G>T, (p.Lys342Asn)  c.742C>T, (p.Arg248Trp) | 13%  16%  16%  15%  46% | none |
| **UM-2** | - | - | - | none |
| **UM-3** | KRAS (NM_033360)  TP53 (NM_000546) | c.35G>T, (p.Gly12Val)  c.1024C>T, (p.Arg342*) | 50%  61% | none |
| **UM-4** | TP53 (NM_000546) | c.743G>A, (p.Arg248Gln) | 32% | none |
| **UM-5** | PIK3CA (NM_006218)  TP53 (NM_000546) | c.1624G>A, (p.Glu542Lys)  c.659A>G, (p.Tyr220Cys) | 20%  37% | KRAS amplification |
| **UM-6** | KDM6A (NM_021140)  TP53 (NM_000546) | c.548_549del, (p.Tyr183*)  c.839G>C, (p.Arg280Thr) | 49%  31% | none |
| **UM-7** | ARID1A (NM_006015)  PIK3CA (NM_006218)  PTEN (NM_000314)  TP53 (NM_000546) | c.325_327delinsTAC, (p.Pro109Leufs*8)  c.3119T>A, (p.Met1040Lys)  c.72C>G, (p.Asp24Glu)  c.524G>A, (p.Arg175His) | 26%  25%  34%  63% | APC deletion  (Exon 16) |
| **UM-8** | APC (NM_000038)  ARID1A (NM_006015)  ARID1A (NM_006015)  CDKN1A (NM_001220777)  KDM6A (NM_021140)  RB1 (NM_000321)  RB1 (NM_000321)  TP53 (NM_000546) | c.1455G>C, (p.Met485Ile)  c.616G>A, (p.Gly206Ser)  c.4805G>A, (p.Ser1602Asn)  c.390_418del, (p.Pro131Lysfs*56)  c.1681G>A, (p.Ala561Thr)  c.533G>T, (p.Ser178Ile)  c.2671C>T, (p.Leu891Phe)  c.1031T>C, (p.Leu344Pro) | 28%  21%  27%  24%  21%  20%  23%  48% | none |
| **UM-9** | ARID1A (NM_006015)  CTNNB1 (NM_001904)  KRAS (NM_033360)  MSH6 (NM_000179)  SMAD4 (NM_005359)  TP53 (NM_000546) | c.2754G>T, (p.Met918Ile)  c.1147T>G, (p.Trp383Gly)  c.436G>A, (p.Ala146Thr)  c.161G>A, (p.Gly54Glu)  c.1128dup, (p.Glu377*)  c.743G>A, (p.Arg248Gln) | 16%  28%  57%  25%  29%  45% | none |
| **UM-10** | KRAS (NM_033360)  TP53 (NM_000546) | c.35G>T, (p.Gly12Val)  c.1024C>T, (p.Arg342*) | 45%  45% | none |
| **UCg-1** | ARID1A (NM_006015)  STAG2 (NM_001042750)  TP53 (NM_000546) | c.5299_5301delinsCTT, (p.Glu1767Leu)  c.499C>T, (p.Gln167Ter)  c.743G>A, (p.Arg248Gln) | 74%  26%  63% | CDKN1A deletion |
| **UCg-2** | KDM6A (NM_021140)  TP53 (NM_000546) | c.1528-1G>C  c.574C>T, (p.Gln192*) | 57%  64% | APC amplification,  CDKN2A deletion  FBXW7 amplification |
| **UCg-3** | TP53 (NM_000546) | c.281C>A, (p.Ser94*) | 50% | none |
| **UCg-4** | KRAS (NM_033360)  PIK3CA (NM_006218) | c.34G>C, (p.Gly12Arg)  c.1633G>A, (p.Glu545Lys) | 28%  18% | none |
| **UCg-5** | ARID1A (NM_006015)  CDKN1A (NM_006015)  CTNNB1 (NM_001904)  STAG2 (NM_001042750)  TP53 (NM_000546) | c.2965C>T, (p.Pro989Ser)  c.419G>A, (p.Arg140Gln)  c.2024C>T, (p.Ser675Leu)  c.913C>T, (p.Arg305*)  c.69G>A, (p.Trp23*) | 14%  16%  16%  80%  56% | none |
| **UCg-6** | APC (NM_000038)  ARID1A (NM_006015)  KDM6A (NM_021140)  PIK3CA (NM_006218)  RB1 (NM_000321)  RB1 (NM_000321)  TP53 (NM_000546) | c.7888G>A, (p.Val2630Ile)  c.5537_5549del, (p.Ile1846Thrfs*33)  c.1663C>T, (p.Gln555*)  c.2176G>A, (p.Glu726Lys)  c.1547G>A, (p.Trp516*)  c.2027dup, (p.Leu676Phefs*16)  c.670G>T, (p.Ser116Cys) | 66%  28%  46%  37%  14%  29%  36% | none |
| **UCg-7** | PIK3CA (NM_006218)  RB1 (NM_000321)  TP53 (NM_000546)  TP53 (NM_000546) | c.263G>A, (p.Arg88Gln)  c.411A>T, (p.Glu137Asp)  c.75-1G>T, p.?  c.347C>G, (p.Ser116Cys) | 35%  37%  19%  31% | RB1 deletion |
| **UCg-8** | CDKN1A (NM_001220777)  CDKN1A (NM_001220777)  CTNNB1 (NM_001904)  PTEN (NM_000314)  RB1 (NM_000321) | c.209dup, (p.Leu71Profs*18)  c.295_296insGGAG, (p.Pro99Argfs*31)  c.1457G>A, (p.Arg486His)  c.217G>A, (p.Glu73Lys)  c.1700C>T, (p.Ser567Leu) | 52%  28%  49%  14%  81% | none |
| **UCg-9** | BRAF (NM_004333)  BRAF (NM_004333)  CDKN1A (NM_001220777)  CDKN2A (NM_000077)  RB1 (NM_000321)  TP53 (NM_000546) | c.1345G>T, (p.Asp449Tyr)  c.1406G>C, (p.Gly469Ala)  c.445G>A, (p.Asp149Asn)  c.178G>A, (p.Ala60Thr)  c.1183C>T, (p.Gln395*)  c.745A>T, (p.Arg249Trp) | 40%  40%  45%  21%  24%  18% | none |
| **UCg-10** | RB1 (NM_000321)  TP53 (NM_000546) | c.1981C>T, (p.Arg661Trp)  c.437G>A, (p.Trp146*) | 31%  37% | none |
| **UCg-11** | PIK3CA (NM_006218)  TP53 (NM_000546) | c.1624G>A, (p.Glu542Lys)  c.659A>G, (p.Tyr220Cys) | 16%  38% | KRAS amplification |
| **CG-1** | - | - | - | none |
| **CG-2** | - | - | - | none |
| **CG-3** | - | - | - | none |
| **IM-1** | FBXW7 (NM_033632) | c.1513C>G, (p.Arg505Gly) | 24% | none |

CG-4-11 and IM-2-8 no NGS data available

**Supplementary Table 3** – SNaPshot^®^ (*FGFR3* and *TERT* Hotspots) and IHC (MSI, SWI/SNF and PD-L1) results

| **Sample name** | **FGFR3**  (R248C, S249C, G372C, S373C, Y375C, G382R, A393E, K652E/M/Q/T) | **TERT**  (-124C>T,  -146C>T**)** | **MSI-IHC**  (MLH1/PMS2, MSH2/MSH6) | **SWI/SNF-IHC**  (INI1/SMARCB1, SMARCA2, SMARCA4, ARID1A, PBRM1) | **PD-L1-IHC**  (TPS/IC-Score/CPS) |
| --- | --- | --- | --- | --- | --- |
| **AE-1** | WT | WT | no loss | no loss | 28-8: 0/0/0  SP142: 0/0/0  SP263: 0/0/0  22C3: na |
| **AE-2** | WT | WT | no loss | no loss | 28-8: 0/0/0  SP142: 0/0/0  SP263: 0/0/0.9  22C3: 0/0/0 |
| **AE-3** | WT | -124C>T | no loss | no loss | 28-8: 0/0/0  SP142: 0/0/0  SP263: 0/1/3  22C3: 0/1/1 |
| **AE-4** | WT | WT | no loss | no loss | 28-8: 0/0/0  SP142: 0/0/0  SP263: 0/0/0  22C3: 0/0/0 |
| **AE-5** | WT | WT | no loss | no loss | 28-8: 0/1/2  SP142: 0/0/0  SP263: 0/2/5  22C3: 0/0/0.9 |
| **AE-6** | WT | -124C>T | no loss | no loss | 28-8: 0/2/5  SP142: 0/0/0  SP263: 0/2/8  22C3: 0/1/3 |
| **AE-7** | WT | WT | no loss | no loss | 28-8: na/0/na  SP142: na/0/na  SP263: na  22C3: na |
| **AE-8** | WT | WT | no loss | ARID1A neg | 28-8: 0/0/0  SP142: 0/0/0  SP263: 0/0/0  22C3: 0/0/0 |
| **AE-9** | WT | WT | no loss | no loss | 28-8: 0/0/0  SP142: 0/0/0  SP263: 0/0/0.9  22C3: 0/0/0 |
| **AM-1** | WT | WT | no loss | no loss | 28-8: 0/2/5  SP142: 0/0/0  SP263: 0/2/7  22C3: 0/1/1 |
| **AM-2** | WT | WT | no loss | no loss | 28-8: 0/0/0  SP142: 0/0/0  SP263: 0/0/0.9  22C3: 0/0/0 |
| **AEM-1** | WT | WT | no loss | no loss | 28-8: 0/0/0  SP142: 0/0/0  SP263: 0/1/0.1  22C3: 0/0/0 |
| **UE-1** | WT | WT | no loss | no loss | 28-8: 0/0/0  SP142: 0/0/0  SP263: 0/0/0  22C3: 0/0/0 |
| **UE-2** | WT | WT | na | na | na |
| **UE-3** | WT | WT | na | na | na |
| **UM-1** | WT | WT | no loss | no loss | 28-8: 0/0/0  SP142: 0/0/0  SP263:0/0/0  22C3: 0/0/0 |
| **UM-2** | WT | WT | no loss | na | na |
| **UM-3** | WT | WT | no loss | na | na |
| **UM-4** | WT | WT | no loss | na | na |
| **UM-5** | WT | WT | no loss | na | na |
| **UM-6** | WT | WT | no loss | na | na |
| **UM-7** | WT | WT | no loss | na | na |
| **UM-8** | WT | WT | no loss | na | na |
| **UM-9** | WT | WT | no loss | na | na |
| **UM-10** | WT | WT | no loss | no loss | 28-8: 0/0/0  SP142: 0/0/0  SP263: 0/0/0  22C3: 0/0/0 |
| **UCg-1** | WT | -124C>T | no loss | no loss | 28-8: 0/0/0.1  SP142: 0/0/0  SP263: 0/0/0  22C3: na |
| **UCg-2** | WT | -124C>T | no loss | no loss | 28-8: 0/0/1  SP142: 0/0/0  SP263: 0/0/0.9  22C3: 0/0/0.9 |
| **UCg-3** | WT | WT | no loss | no loss | 28-8: 0/0/0  SP142: 0/0/0  SP263: 0/0/0.9  22C3: 0/0/0 |
| **UCg-4** | WT | WT | no loss | no loss | 28-8: 0/1/4  SP142: 0/0/0  SP263: 0/2/7  22C3: 0/0/0.9 |
| **UCg-5** | WT | -124C>T | no loss | no loss | 28-8: 0/0/0  SP142: 0/0/0  SP263: 0/0/0.9  22C3: 0/0/0.9 |
| **UCg-6** | WT | -124C>T | no loss | no loss | 28-8: 0/2/7  SP142: 0/0/0  SP263: 0/3/20  22C3: 0/2/8 |
| **UCg-7** | WT | -124C>T | no loss | no loss | 28-8: 0/2/5  SP142: 0/0/0  SP263: 0/3/25  22C3: 0/1/1 |
| **UCg-8** | WT | -124C>T | no loss | no loss | 28-8: 0/0/0  SP142: 0/0/0  SP263: 0/0/0  22C3: 0/0/0 |
| **UCg-9** | WT | -124C>T | no loss | SMARCA2 neg | 28-8: 0/0/0  SP142: 0/0/0  SP263: 0/0/0  22C3: 0/0/0 |
| **UCg-10** | WT | WT | na | na | na |
| **UCg-11** | WT | WT | no loss | PBRM1 neg | 28-8: 0/0/0  SP142: 0/0/0  SP263: 0/0/0.9  22C3: 0/0/0.9 |
| **CG-1** | WT | WT | na | na | na |
| **CG-2** | WT | WT | na | na | na |
| **CG-3** | WT | WT | na | na | na |
| **CG-4** | na | WT | na | na | na |
| **CG-5** | na | WT | na | na | na |
| **CG-6** | na | WT | na | na | na |
| **CG-7** | na | WT | na | na | na |
| **CG-8** | na | WT | na | na | na |
| **CG-9** | na | WT | na | na | na |
| **CG-10** | na | WT | na | na | na |
| **CG-11** | na | WT | na | na | na |
| **IM-1** | WT | -124C>T | na | na | na |
| **IM-2** | na | WT | na | na | na |
| **IM-3** | na | WT | na | na | na |
| **IM-4** | na | WT | na | na | na |
| **IM-5** | na | WT | na | na | na |
| **IM-6** | na | WT | na | na | na |
| **IM-7** | na | WT | na | na | na |
| **IM-8** | na | WT | na | na | na |

na=not available, TPS=Tumour cell proportion score/Cologne Score, IC-Score=Immune cell score, CPS=combined positivity score
