# Supplementary material for: Comparative genomic profiling of glandular bladder tumours
Source: Virchows Arch. 2020 Mar 20;477(3):445–54. doi: 10.1007/s00428-020-02787-8 (PMC7443184; doi:10.1007/s00428-020-02787-8)
Supplement: Supplementary file 3 — (DOCX 508 kb) [file 428_2020_2787_MOESM3_ESM.docx]

**Online Resource 3 – Virchows Archiv**

**Supplementary Figures 1-2**

**Comparative genomic profiling of glandular bladder tumours**

Angela Maurer^a^, Nadina Ortiz-Bruechle^a^, Karolina Guricova^a^, Michael Rose^a^, Ronja Morsch^a,b^, Stefan Garczyk^a^, Robert Stöhr^c^, Simone Bertz^c^, Reinhard Golz^d^, Henning Reis^e^, Felix Bremmer^f^, Annette Zimpfer^g^, Sabine Siegert^h^, Glen Kristiansen^i^, Kristina Schwamborn^j^, Nikolaus Gassler^k,l^, Ruth Knuechel^a^, Nadine T. Gaisa^a^ for the German study group of bladder cancer

^a^ Institute of Pathology, RWTH Aachen University, Aachen, Germany

^b^ Department of Urology, RWTH Aachen University, Aachen, Germany

^c^ Institute of Pathology, University Hospital Erlangen, Erlangen, Germany

^d^ Institute of Pathology, HELIOS Clinic Wuppertal, Wuppertal, Germany

^e^ Institute of Pathology, University Hospital Essen, University of Duisburg-Essen, Germany

^f^ Institute of Pathology, University Medical Center, University of Göttingen, Göttingen, Germany

^g^ Institute of Pathology, University Medical Center Rostock, Rostock, Germany

^h^ Institute of Pathology Munich-North, Munich, Germany

^i^ Institute of Pathology, University Hospital Bonn, Bonn, Germany

^j^ Institute of Pathology, Technical University Munich, Munich, Germany

^k^ Institute of Pathology, Hospital Braunschweig, Braunschweig, Germany

^l^ Pathology, University Hospital Jena, Jena, Germany

**Corresponding author:**

Nadine T. Gaisa, MD, PhD; ORCID 0000-0002-4762-3964

Institute of Pathology, RWTH Aachen University

Pauwelsstrasse 30

52074 Aachen, Germany

Phone: +49-241-8089288; Fax: +49-241-8082439; email: [ngaisa@ukaachen.de](mailto:ngaisa@ukaachen.de)


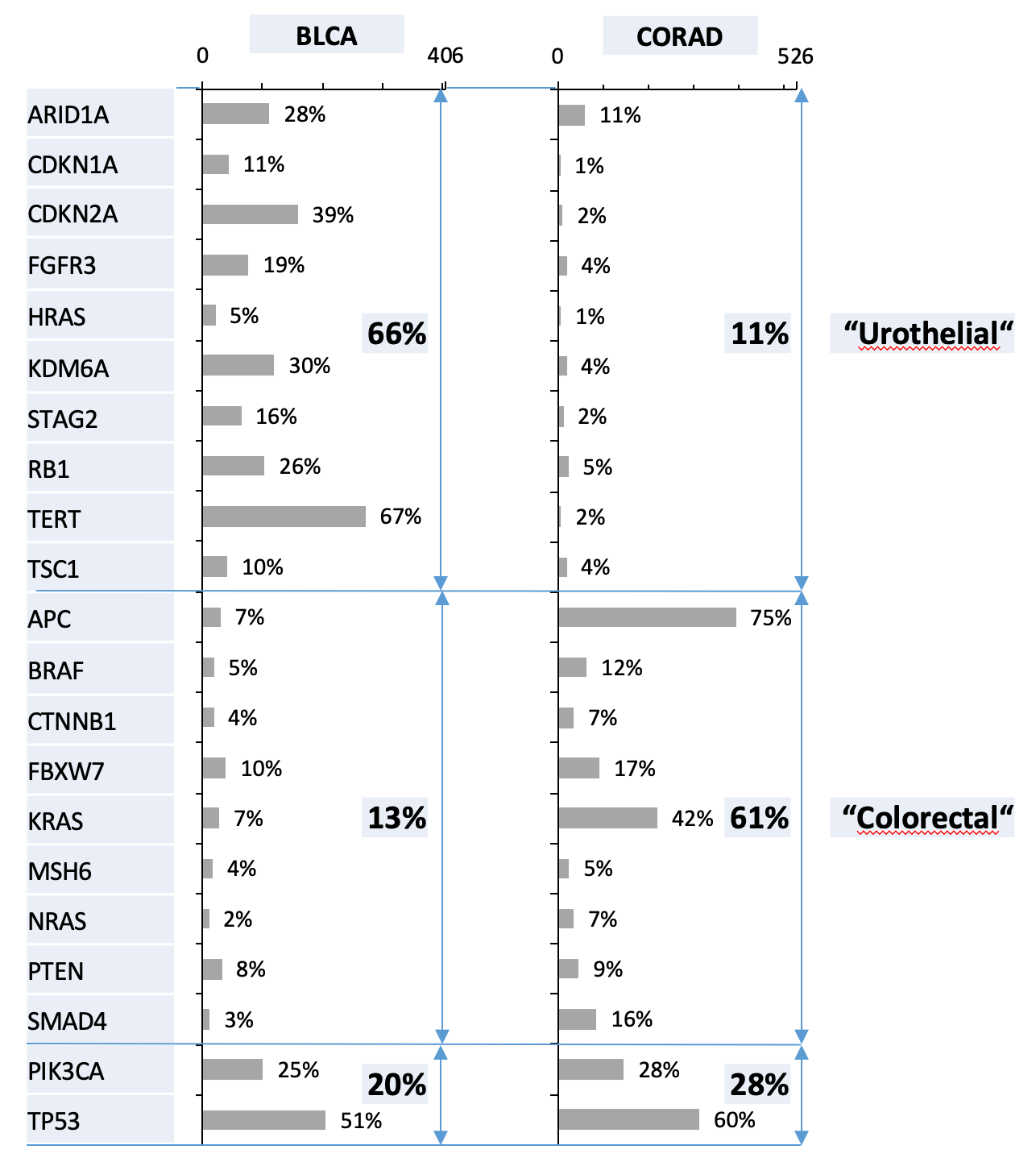


**Supplementary Figure 1: Genomic alterations in muscle invasive bladder cancer (BLCA) and colorectal adenocarcinoma (CORAD)**

Individual alteration frequencies for 21 genes used for the cumulative frequency calculation for alterations of urothelial or colorectal associated genes. Data for BLCA and CORAD alterations for the 21 genes was obtained from The Cancer Genome Atlas Research Network (TCGA) pan-cancer analysis project (accessed through http://cbioportal.org, [1]).


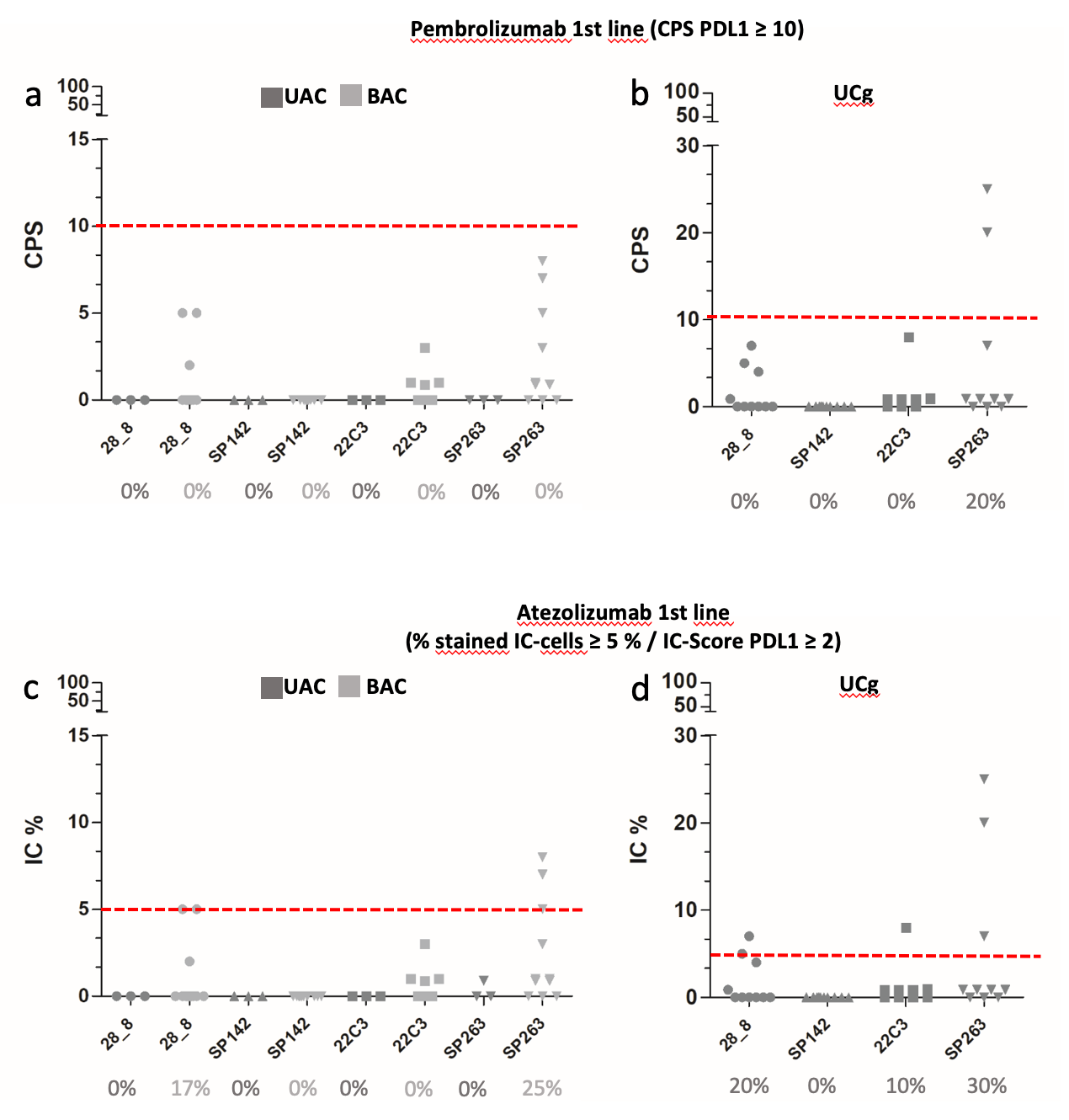


**Supplementary Figure 2: PD-L1 expression in glandular bladder tumours and potential relevance for checkpoint inhibitor therapy**

Four different anti-PD-L1 antibodies (28-8, SP142, SP263, 22C3) were used for immunohistochemical PD-L1 expression analysis of glandular bladder tumours (n=12 primary bladder adenocarcinoma (BAC), n=3 urachal adenocarcinoma (UAC) and n=10 urothelial carcinoma with glandular differentiation (UCg)). TPS (proportional staining of tumour cells), IC-Score (staining of immune cells) and CPS (calculated combined positivity score) were determined. (A) and (B) CPS for BAC, UAC and UCg for all samples and all four antibodies. Threshold for 1^st^ line Pembrolizumab therapy in advanced bladder cancer (CPS-Score ≥ 10) is marked with a dotted red line. (C) and (D) % stained IC-cells for BAC, UAC and UCg for all samples and all four antibodies. Threshold for 1^st^ line Atezolizumab therapy in advanced bladder cancer (≥ 5% stained cells / IC-Score ≥ 2) is marked with a dotted red line.

**References Supplementary Figures 1-2:**

[1] The Cancer Genome Atlas Research Network, Weinstein JN, Collisson EA, Mills GB, Mills Shaw KR, Ozenberger BA, Ellrott K, Shmulevich I, Sander C, Stuart JM (2013) The Cancer Genome Atlas Pan-Cancer analysis project. Nature Genetics 45:1113-1120. https://doi.org/10.1038/ng.2764
